# Déficit congénital en facteur VII révélé par une hémorragie post circoncision

**DOI:** 10.11604/pamj.2019.33.212.13226

**Published:** 2019-07-16

**Authors:** Mariem Ettarfaoui, Salma El Ghissassi, Amina Barkat

**Affiliations:** 1 Service de Médecine et Réanimation Néonatale, PV, HER, CHU Ibn Sina, Commission de Formation Médicale Continue Université Mohammed V, Faculté de Médecine et Pharmacie, Maroc; 2 Équipe de Recherche en Santé et Nutrition du Couple Mère Enfant, Maroc

**Keywords:** Facteur VII, déficit, congénital, circoncision, Factor VII, deficit, congenital, circumcision

## Abstract

Le facteur VII ou proconvertine est un facteur de la coagulation vitamine K-dépendant qui intervient dans la voie exogène de la coagulation. Le déficit constitutionnel en facteur VII est une maladie héréditaire de transmission autosomique récessive, très rare. Nous rapportons le cas d'un nouveau-né issu de parents consanguins du premier degré, et admis à J10 de vie pour prise en charge d'un syndrome hémorragique post circoncision. Le bilan biologique a montré un taux de prothrombine bas et un TCA normal. Un bilan de la coagulation a montré un déficit isolé en facteur VII évalué à 18%. Le nouveau-né a été transfusé de culots globulaires et de plasma frais congelé permettant d'arrêter l'hémorragie avec une évolution favorable. A travers cette observation, nous rappelons les caractéristiques de ce déficit rare. Le pronostic de la maladie reste lié au risque de survenue d'hémorragies graves, notamment cérébrales survenant en période néonatale comme c'est le cas de notre patient. D'où la nécessité de faire un bilan de crase pré-circoncision.

## Abstract (en)

Factor VII formerly known as proconvertin is a vitamin K-dependent coagulation factor involved in exogenous coagulation. Congenital factor VII deficiency is a very rare autosomal recessive hereditary disease. We report the case of a new-born of parents who had first-degree consanguinity, admitted to hospital on D10 of life with post-circumcision haemorrhagic syndrome. Laboratory tests showed decreased levels of prothrombin and normal activated partial thromboplastin time. Coagulation tests showed isolated factor VII deficiency to 18%. The new-born received transfusion of packed red blood cells and fresh frozen plasma in order to stop bleeding with favorable outcome. This study highlights the features of this rare deficit. Prognosis is linked to the risk of severe bleedings, such as brain bleeding occurring in the neonatal period as is the case with our patient. Hence the need to perform blood crasis assessment before circumcision.

## Introduction

Le facteur VII de la coagulation ou proconvertine est une glycoprotéine du sang, synthétisée par le foie. Ce facteur est vitamine K-dépendant. Le déficit en facteur VII ou maladie d'Alexander est une affection très rare, se manifestant par un syndrome hémorragique d'expression variable. Le tableau clinique peut être très sévère avec la survenue précoce d'hémorragies intracérébrales, ou d'hémarthroses à répétition, ou au contraire modéré avec des hémorragies cutanéo-muqueuses ou des hémorragies provoquées par une intervention chirurgicale [1, 2]. Nous rapportons dans notre observation une révélation néonatale d'un déficit en facteur VII suite à une hémorragie post circoncision.

## Patient et observation

Nous rapportons le cas du nouveau-né E.A âgé de 10 jours, de parents consanguins de premier degré, mère âgée de 43 ans ayant un groupage A positif, G2P2. La grossesse était suivie menée à terme, l'accouchement était par voie haute avec une présentation de siège et un Apgar à 10/10, sans notion d'hémorragie à la chute du cordon. Un frère âgé de 3 ans bien portant circoncis à J15 de vie sans incidents, pas de cas similaires dans la famille. Le nouveau-né a été circoncis à J10 de vie, traditionnellement à domicile, sans aucun bilan préalable, et a présenté un syndrome hémorragique post circoncision qui a nécessité son admission au service de néonatologie. L'examen à l'admission avait objectivé un nouveau-né pâle hypotonique avec des conjonctives décolorées, stable sur le plan hémodynamique, neurologique et respiratoire, le reste de l'examen somatique était sans particularités. Un bilan réalisé a souligné une anémie normochrome normocytaire avec une concentration en hémoglobine à 7,1 g/dl et un taux de plaquettes normal à 165 000 plaquettes/mm^3^. Le bilan d'hémostase révélait un taux de prothrombine (TP) à 30%, avec un temps de céphaline activée (TCA) à 32 s et un taux de fibrinogène normal. Compte tenu de la consanguinité parentale, du syndrome hémorragique avec un TP bas et un TCA normal, un déficit en facteur VII était suspecté. Le dosage a confirmé le diagnostic en montrant un taux initial en facteur VII à 18%. Ces dosages avaient été effectués avant tout traitement substitutif, en période hémorragique. Le dosage chez les parents a montré des taux modérément diminués 38% chez la mère et 51% chez le père en faveur d'un statut d'hétérozygote. Un bilan radiologique fait d'une échographie trans-fontannellaire et d'une échographie abdomnio-rénale est revenu normal. Le nouveau-né a été transfusé de culots globulaires et de plasma frais congelé permettant d'arrêter l'hémorragie avec une évolution favorable et un examen normal à la sortie. Les parents ont été informés de la nécessité de la mise en œuvre d'un traitement substitutif en cas de risque hémorragique.

## Discussion

Le déficit congénital en facteur VII a été décrit pour la première fois en 1951 par Alexander. Sa prévalence est estimée à 1/ 1.000.000 [3]. Il est transmis selon le mode autosomique récessif, d'où sa fréquence dans les mariages consanguins, ce qui est le cas de notre patient issu d'un mariage consanguin au premier degré. Seuls les patients homozygotes ou hétérozygotes composites peuvent présenter un syndrome hémorragique, les sujets hétérozygotes sont asymptomatiques. Le gène responsable est localisé sur le chromosome 13 [1]. Il existe deux types de déficits en facteur VII [4, 5]: un déficit congénital transmis par les parents et un déficit acquis secondaire à d'autres affections (affections malignes, hépatopathies, certains médicaments…). Il n'existe pas de corrélation entre la sévérité du saignement et l'importance du déficit, et la symptomatologie est variable [1, 6]. Trois paliers de gravité ont cependant été distingués suivant la profondeur du déficit: taux de facteur VII inférieur à 5%, entre 5 et 20%, et supérieur à 20%. Le diagnostic positif repose sur des éléments biologiques [4-7] notamment un temps de Quick allongé évoquant une anomalie de la voie exogène; et un TCK normal assurant une intégrité de la voie endogène. La confirmation est apportée par le dosage des facteurs de coagulation (VII, II, X et fibrinogène). L'enquête familiale est essentielle, notamment le dosage du facteur VII chez les parents et la fratrie du sujet atteint. Le traitement est indiqué en cas d'accident hémorragique aigu, et repose sur l'administration du plasma frais congelé à raison de 10 à 15 ml/kg, qui peut être efficace, mais qui présente certains inconvénients: le PFC renferme relativement peu de facteur VII, et il faut en administrer beaucoup pour fournir la quantité requise de facteur VII, sans compter le risque de transmission des virus (hépatite à virus B [HVB], hépatite à virus C HVC]). L'administration du concentré PPSB est de moins en moins utilisée du fait du risque élevé de complications thromboemboliques [5]. Par contre, l'administration de concentré de facteur VII, est efficace en sachant que 0,5 U/kg fait augmenter le taux de proconvertine de 1% mais reste non disponible dans notre contexte.

Lorsqu'une intervention chirurgicale est programmée, des injections prophylactiques sont indiquées: 20 à 30 mg/kg de facteur VII sont administrés en préopératoire et 5 à 10 mg/kg toutes les 4 à 6h en postopératoire pendant 5 à 10 j [8, 9]. En cas de saignements graves et répétitifs, un traitement au long cours par 2 injections par semaine a été proposé par certains auteurs [2]. Dans le cas de notre observation, il s'agit d'un déficit en facteur VII découvert en période néonatale chez un nouveau-né de parents consanguins présentant le déficit à l'état hétérozygote et compliqué d'une hémorragie post circoncision. Le déficit en facteur VII se traduit par un syndrome hémorragique qui reste le maître symptôme et s'observe chez les sujets homozygotes comme c'est le cas de notre patient. Un sujet hétérozygote n'en est que très rarement affecté c'est le cas des parents de notre patiente qui n'ont jamais présenté de syndrome hémorragique. Le taux de facteur VII chez notre patient est de 18% correspondant ainsi au palier compris entre 5 à 20% de la normale, dans cette catégorie les saignements sont en général modérés. Mais paradoxalement, notre patient a présenté un tableau sévère, confirmant ainsi le fait que la sévérité du saignement ne semble pas liée à l'importance du déficit. Dans le cas de notre patient il n'a pas été proposé de traitement au long cours néanmoins les parents ont été mis en garde du risque de récidives et informés de la nécessité de la mise en œuvre d'un traitement substitutif en cas de risque hémorragique.

## Conclusion

Le déficit en facteur VII est une affection héréditaire rare. Toutefois, dans sa forme grave, elle peut mettre en jeu le pronostic fonctionnel, voire vital. Le pronostic de la maladie reste lié au risque de survenue d'hémorragies graves, notamment cérébrales survenant en période néonatale comme c'est le cas de notre patiente. D'où la règle de ne faire de circoncision qu'après bilan de crase.

## Conflits d’intérêts

Les auteurs ne déclarent aucun conflit d'intérêts.

## References

[1] Giansily-Blaizot M, Verdier R, Biron-Adréani C, Schved JF, Bertrand MA, Borg JY (2004). Analysis of biological phenotypes from 42 patients with inherited factor VII deficiency: can biological tests predict the bleeding risk. Haematologica.

[2] Tafer N, Julliac B, Morel N, Dabadie P (2007). Hémorragie sous arachnoidienne par rupture d'anévrisme et déficit en facteur VII, à propos d'un cas. Annales Françaises d'Anesthésie et de Réanimation.

[3] Sfaihi Ben Mansour L, Thabet A, Aloulou H, Turki H, Chabchoub I, Mhiri F (2009). Factor VII deficiency revealed by intracranial hemorrhage. Arch Pédiatr.

[4] Imane Z, Mdaghri-Alaoui A, Hamdani S, El Harim-Lamdouar L, Lamdouar-Bouazzaoui N (2004). The constitutional deficiency in factor VII: about one case. J PédiatrPuériculture.

[5] Boltin D, Boguslavski V, Goor Y, Elkayam O (2008). Primary factor VII deficiency. Isr Med Assoc J.

[6] Giansily-Blaizot M, Biron-Andreani C, Aguilar-Martinez P, de Moeloose P, Briquel ME, Goudemand J (2002). Inherited factor VII deficiency and surgery: clinical data are the best criteria to predict the risk of bleeding. Br J Haematol.

[7] Hedner U (2006). Mechanism of action of recombinant activated factor VII: an update. Semin Hematol.

[8] Boffard KD, Riou B, Warren B, Choong PI, Rizoli S, Rossaint R (2005). Recombinant factor VIIa as adjunctive therapy for bleeding control in severely injured trauma patients. Two parallel randomized, placebocontrolled, double-blind clinical trials. J Trauma.

[9] Lecompte T, Toussaint-Hacquard M, Carteaux JP (2006). Eptacogalpha (activé) ou facteur VII de la coagulation, humain recombinant et activé, en chirurgie cardiaque avec CEC: le point en.

